# Comparison of Dual-Energy X-ray Absorptiometry (DXA) Versus a Multi-Frequency Bioelectrical Impedance (InBody 770) Device for Body Composition Assessment after a 4-Week Hypoenergetic Diet

**DOI:** 10.3390/jfmk4020023

**Published:** 2019-04-25

**Authors:** Jose Antonio, Madaline Kenyon, Anya Ellerbroek, Cassandra Carson, Victoria Burgess, Denvyr Tyler-Palmer, Jonathan Mike, Justin Roberts, Gerseli Angeli, Corey Peacock

**Affiliations:** 1 Exercise and Sport Science, Nova Southeastern University, Davie, FL 33328, USA; 2 Exercise Science, Grand Canyon University, Phoenix, AZ 85017, USA; 3 Cambridge Centre for Sport and Exercise Sciences, Anglia Ruskin University, Cambridge CB1 1PT, UK; 4 Rua Botucatú 740-Edif. Octávio de Carvalho, 04023-900 São Paulo, Brazil

**Keywords:** exercise, fat mass, diet, body composition, fat-free mass, exercise-trained

## Abstract

The purpose of this investigation was to compare two different methods of assessing body composition (i.e., a multi-frequency bioelectrical impedance analysis (MF-BIA) and dual-energy x-ray absorptiometry (DXA)) over a four-week treatment period in exercise-trained men and women. Subjects were instructed to reduce their energy intake while maintaining the same exercise regimen for a period of four weeks. Pre and post assessments for body composition (i.e., fat-free mass, fat mass, percent body fat) were determined via the MF-BIA and DXA. On average, subjects reduced their energy intake by ~18 percent. The MF-BIA underestimated fat mass and percentage body fat and overestimated fat-free mass in comparison to the DXA. However, when assessing the change in fat mass, fat-free mass or percent body fat, there were no statistically significant differences between the MF-BIA vs. DXA. Overall, the change in percent body fat using the DXA vs. the MF-BIA was −1.3 ± 0.9 and −1.4 ± 1.8, respectively. Our data suggest that when tracking body composition over a period of four weeks, the MF-BIA may be a viable alternative to the DXA in exercise-trained men and women.

## 1. Introduction

Body composition assessments are an important part of determining the effects of various interventions related to diet, training, and supplementation [1,2,3,4,5,6,7,8]. Numerous methods exist for assessing body composition, which attempt to accurately estimate fat-free mass (FFM) and fat-mass (FM), and their subcomponents. FFM can be further classified into components that include skeletal muscle mass, bone, and body water. Early work by Wang et al. [9,10] proposed a five-level model for organizing body composition compartments and since then has been divided into additional compartments to include two (2C), three (3C) and four (4C) compartments. While multi-compartmental models have established reputations as being the “gold standard,” they do possess limitations. Therefore, more commonly used assessments are used including hydrodensitometry (underwater weighing), air displacement plethysmography (BOD POD^®^), skinfold thickness, and bioelectrical impedance analysis (BIA). Although all body composition assessments are subject to limitations and confounding influences, dual energy x-ray absorptiometry (DXA) measures bone mineral content (BMC), fat mass, and fat-free mass and is often used as a reference method for estimating body composition [11,12,13]. However, limitations do exist as the DXA is non-portable, costly, and often necessitates training by a licensed technician due to small amounts of radiation exposure.

Bioelectrical impedance analysis has evolved to include the use of multiple frequencies and impedance measurements to improve the accuracy and reliability of body composition estimates [14]. Compared with other methods, BIA is relatively simple, quick, and noninvasive [15,16,17]. Unlike underwater weighing, bioelectrical impedance analysis does not require total body submersion in water. Moreover, there is no exposure to radiation as found with a typical DXA scan. It should be noted that the various BIA devices differ in the number, type, and placement of electrodes and this can certainly affect body composition assessments [18]. In addition, multi-frequency BIA machines (e.g., InBody 770) have been developed for assessing segmental and total body composition [17,19]. Recent studies suggest multi-frequency devices may be less subject to error caused by redistribution of total body water between extracellular water and intracellular water and likely a superior method for the estimation of total body composition [20].

Prior studies using MF-BIA have demonstrated suitable estimates of body composition, despite underestimating fat mass and overestimating fat-free mass when compared to DXA [21,22,23]. On the other hand, one investigation showed that MF-BIA is inferior to DXA in tracking changes in body composition in response to endurance training, strength training, or concurrent training in middle-aged older women [24]. Accurate assessment of body composition plays a role in health, athletic performance and is often critical during for physique competitors. In fact, the assessment of percent body fat is crucial in sports (e.g., physique shows) that require competitors to decrease energy intake so as to achieve a leaner physique. Therefore, the purpose of this study was to compare the use of dual-energy x-ray absorptiometry (DXA) to an MF-BIA device (InBody 770) for body composition assessment after a four-week hypoenergetic diet in exercise-trained men and women.

## 2. Materials and Methods

### 2.1. Participants

Forty-one exercise-trained men and women (29 women, 12 men) volunteered for this investigation. We recruited a convenience sample of subjects that at minimum had been training regularly for at least one year with a frequency of three times per week. These subjects were from the community as well as students or staff from the University. Sedentary subjects were excluded from the trial. Research participants came to the laboratory on two occasions for testing (baseline and four weeks). In accordance with the Helsinki Declaration, the University’s Institutional Review Board approved all procedures involving human subjects. Written informed consent was obtained prior to participation (IRB# 2018-232-NSU, approved 14 June 2018).

### 2.2. Body Composition

Body composition was assessed with a dual-energy X-ray absorptiometry machine (DXA) (Model: Hologic Horizon W, Hologic Inc., Danbury, CT, USA) and an InBody 770 multi-frequency bioelectrical impedance (BIA) device (InBody 770, Cerritos, CA, USA). Participants were instructed to come to the laboratory after at least a three-hour fast and no prior exercise that day. All testing was performed between 1130 and 1400. Subjects typically came to the lab at the same time for each of the testing dates. For the DXA, quality control calibration procedures were performed on a spine phantom. Subjects had their weight determined on a calibrated scale. Subjects wore typical athletic clothing and removed all metal jewelry. They were positioned supine on the DXA within the borders delineated by the scanning table. Each whole body scan took approximately seven minutes. For the InBody 770 MF-BIA, subjects stood on the platform of the device barefoot with the soles of their feet on the electrodes. Subjects then grasped the handles of the unit with their thumb and fingers to maintain direct contact with the electrodes. They stood still for ~1 min while maintaining their elbows fully extended and their shoulder joint abducted to approximately a 30-degree angle. 

### 2.3. Diet and Exercise

Participants were instructed to reduce their energy intake by ~20–25% via the reduction in carbohydrate and fat intake during the treatment period. Our pool of subjects typically logged their food on a regular basis. Thus, they were well versed at tracking their intake. Subjects were also instructed initially to provide a baseline food log prior to testing (24-h recall). Subsequently, they were instructed to keep a food log (three times per week, two weekdays, one weekend day) on the MyFitnessPal mobile app [25] for the duration of the treatment period. The subjects had prior experience using the mobile app and as such were quite skilled at logging their food intake. The subjects’ training regimen was not altered (i.e., this was not a training study). Thus, each subject self-selected their exercise regimen. In order to ascertain their training regimen, subjects filled out a questionnaire (i.e., average hours of training per week and the number of years of training regularly). In addition, subjects were asked to keep their protein intake at their current levels or higher. We provided protein powder (VPX, Weston, FL, USA) to subjects in the event that they needed assistance in complying with the dietary protein requirements.

### 2.4. Statistical Analysis

A paired *t*-test was used to analyze differences between the DXA and BIA. All data is presented in the tables are the mean ± SD. Data presented in the figures are the mean and 95% confidence interval. GraphPad (Prism 6) software was used for statistical analyses.

## 3. Results

The subjects’ physical characteristics are shown in Table 1. 

On average, subjects reduced their energy intake by ~18%. This reduction was accomplished via a significant decrease in carbohydrate and fat intake. Although there was a statistically significant increase in protein intake per unit body weight, the increase in protein intake (~10 g per day) was rather minor (Table 2).

After the four-week treatment, there was a significant decrease in body weight, fat mass and percent body fat as determined by both the DXA and BIA. However, there were no changes in fat-free mass. The BIA underestimated fat mass and percent body fat in comparison to the DXA. In addition, the BIA overestimated fat-free mass versus the DXA (Table 3). On the other hand, the change (post minus pre) in body weight, fat-free mass, fat mass, and percent body fat were not different between the DXA and BIA (Table 3). 

**Figure 1:**
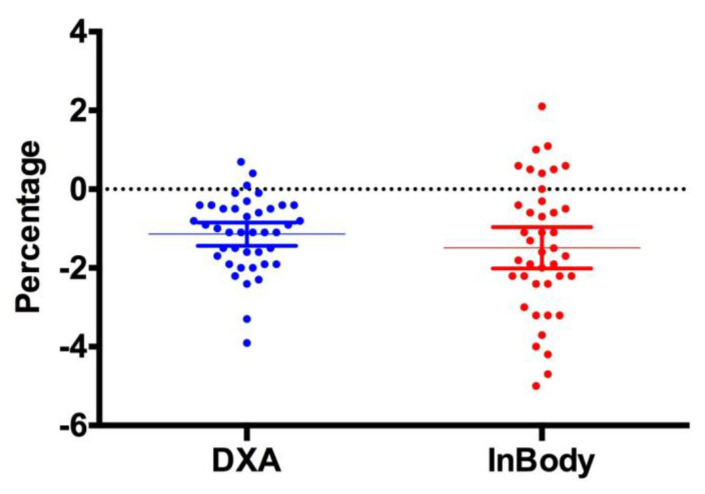
Change in Body Fat Percent. This figure shows individual data points comparing the DXA vs. InBody BIA. The data is presented as the mean (middle horizontal line) with a 95% confidence interval (CI). DXA: Lower 95% CI of mean −1.437, Upper 95% CI of mean −0.8457; InBody: Lower CI of mean −2.016, Upper CI of mean −0.9641.

**Figure 2:**
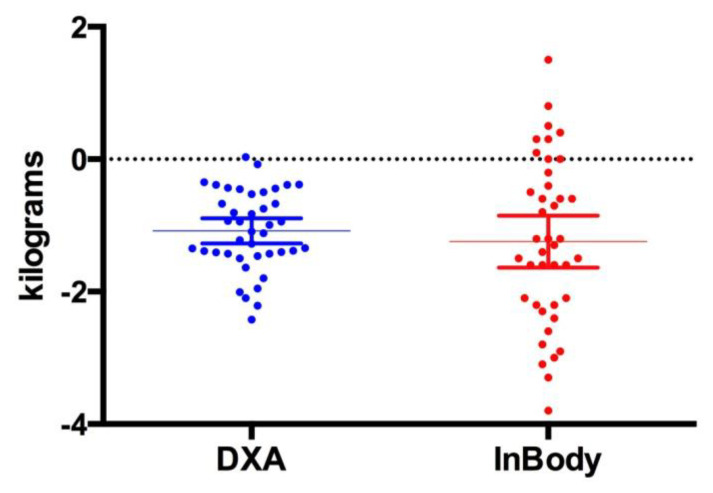
Change in Fat Mass. This figure shows individual data points comparing the DXA vs. InBody BIA. The data is presented as the mean (middle horizontal line) with a 95% confidence interval (CI). DXA: Lower 95% CI of mean −1.272, Upper 95% CI of mean −0.8924, InBody: Lower CI of mean −1.637, Upper CI of mean −0.8512.

**Figure 3:**
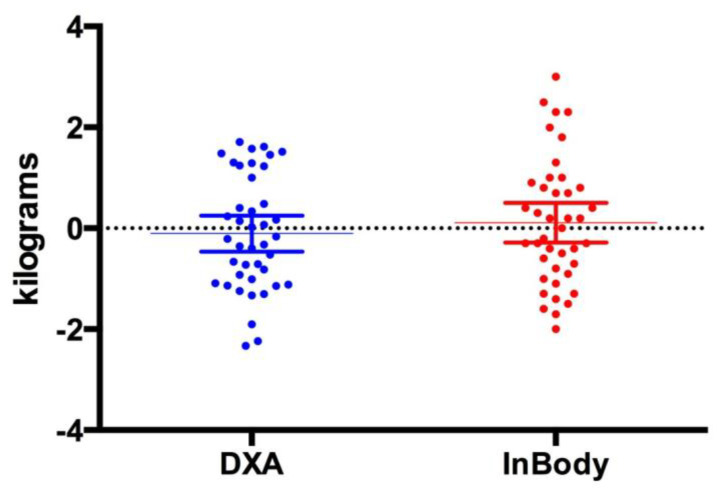
Change in Fat-free mass. This figure shows individual data points comparing the DXA vs. InBody BIA. The data is presented as the mean (middle horizontal line) with a 95% confidence interval (CI). DXA: Lower 95% CI of mean −0.4627, Upper 95% CI of mean 0.2505, InBody: Lower CI of mean –0.2841, Upper CI of mean 0.5037.

**Figure 4:**
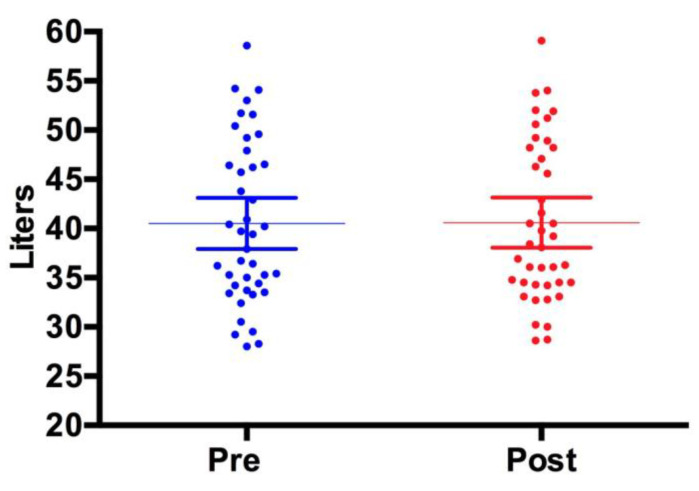
Total Body Water. The InBody BIA device was used to assess total body water. There were no changes pre versus post for total body water. The data is presented as the mean (middle horizontal line) with a 95% confidence interval (CI). Pre: Lower 95% CI of mean 37.91, Upper 95% CI of mean 43.12. Post: Lower CI of mean 38.05, Upper CI of mean 43.15.

Figure 1, Figure 2 and Figure 3 show individual data points when comparing the DXA versus the InBody BIA. Despite the fact that there were no statistically significant differences in the change in fat mass, fat-free mass or percent body fat, it should be noted that the BIA showed a high degree of variability (i.e., greater standard deviation) compared to the DXA. Total body water did not change pre (40.5 ± 8.3 L) versus post (40.6 ± 8.1 L, *p* = 0.5484) (Figure 4).

## 4. Discussion

The primary finding from the current investigation is that the MF-BIA device (InBody 770) provides data (i.e., changes in body composition) comparable to the DXA. When comparing actual fat-free mass and fat mass, the MF-BIA overestimates the former and underestimates the latter. In spite of the fact that the MF-BIA predicted changes in body composition (i.e., fat mass and fat-free mass) in a manner similar to the DXA, it is apparent that the MF-BIA demonstrates a much higher degree of variability. Nonetheless, most athletes and coaches are particularly interested in tracking the change in body composition and not necessarily the absolute values. That is, an absolute value for any measure of body composition (i.e., fat mass, fat-free mass, or percent body fat) is unimportant for sports performance. Coaches typically want to see a directional change (i.e., is fat mass decreasing or fat-free mass increasing). Body composition values in and of itself do not predict sports performance.

Previous research has compared various types of BIA devices with DXA with variable results depending on the type of BIA device used. Schoenfeld et al. assessed multi-frequency bioelectrical impedance versus DXA in young male volunteers after a 10-week training program [17]. The resistance training program included exercises for all major muscle groups. They discovered that the mean changes were not significantly different when comparing MF-BIA with DXA for percent body fat (−1.05 vs. −1.28%), fat mass (−1.13 vs. −1.19 kg), and fat-free mass (0.10 vs. 0.37 kg, respectively). Interestingly, the percent body fat changes in our investigation (DXA −1.1% and MF-BIA −1.5%) are similar to that of Schoenfeld et al. Moreover, both investigations used exercise-trained subjects. Collectively these studies suggest that MF-BIA is a viable alternative for tracking changes in fat mass and fat-free mass during a combined diet and exercise program in young, athletic men or in a hypocaloric state in exercise-trained men and women [17].

Other studies have examined exercise-trained populations. Scientists compared MF-BIA (Inbody 720) and DXA for assessing body composition in 45 collegiate female athletes [26]. The InBody underestimated percent body fat and overestimated fat-free mass in comparison to the DXA. However, the InBody 720 and DXA both measures were similar with regards to total body and segmental lean soft tissue. [26]. In 43 older women (65 years) that resistance-trained three times per week for three months (i.e., two sets of eight exercises 10–15 repetitions) they found that single-frequency BIA-derived equations may not provide sufficient accuracy to track changes in fat-free mass after 12 weeks of resistance training in older women [27].

There is a plethora of cross-sectional studies that have compared various BIA devices with the DXA. According to Karelis et al., the Inbody 230 is an acceptable device to measure fat mass, percent body fat, and total fat-free mass (except for women) in healthy adults [28]. The InBody 520 and 720 are valid estimators of lean body mass and fat mass in men and of lean body mass, appendicular lean mass, and fat mass in women. Moreover, the 720 and 520 are valid estimators of trunk lean mass in men and women, respectively [29]. Furthermore, MF-BIA and DXA were comparable for assessing body composition in populations with higher body fat percentages [19]. However, others have shown that BIA underestimates body fat percentage and fat mass while overestimating lean body mass, compared with DXA [30].

Although not a primary focus of this investigation, it is noteworthy that fat-free mass did not decrease as a result of caloric restriction. Piatti et al. found that a hypocaloric diet providing a high percentage of protein can improve insulin sensitivity as well as spare lean body mass [31]. Moreover, a high protein diet was superior to a high carbohydrate diet with regards to improving body composition in overweight and obese males [32]. Thus, the fact that participants in our study maintained a high protein intake coupled with their regular exercise habits contributed to the sparing of fat-free mass. Furthermore, the maintenance of fat-free mass was not due to an elevation in total body water in as much as that value did not change over the four-week treatment period. It is noteworthy to mention the limitations of this investigation. First, the sample of relatively young exercise-trained men and women warrants caution vis-à-vis its application to other populations (e.g., youth and older adults). Another potential limitation is the use of the DXA as a criterion reference method. According to Moon et al., DXA may overestimate percent body fat up to 3.7% when compared to a criterion five-compartment model [33]. Nevertheless, the DXA is often used as a criterion method. Thus, it would behoove investigators to understand the limitations of this as well as other body composition assessment methods.

## 5. Conclusions

The data from this investigation demonstrate that an MF-BIA device (InBody 770) can predict changes in body composition (i.e., fat mass, fat-free mass, and percent body fat) similar to a DXA. It should be noted that this applies to group changes and that in general, the MF-BIA device shows more variability than the DXA.

## Figures and Tables

**Table 1:** Physical Characteristics and Training History.

**Age (years)**	33 ± 10
Height (centimeters)	169 ± 8
Average hours of aerobic training per week	5.3 ± 4.2
Average hours of resistance training per week	4.2 ± 2.7
Average hours of training of other non-traditional exercise (e.g., pilates, yoga)	2.4 ± 4.2
Average years of exercise training	13.3 ± 6.1

Data are expressed as the mean ± SD. *n* = 41 (*n* = 29 women, *n* = 12 men).

**Table 2:** Energy and Macronutrient Intake.

	Pre	Post	*p*-Value Pre vs. Post
Energy (kcal)	1943 ± 555	1580 ± 429	<0.0001
Protein (g)	130 ± 49	140 ± 43	0.0907
Carbohydrate (g)	202 ± 73	140 ± 64	<0.0001
Fat (g)	68 ± 24	51 ± 18	<0.0001
Energy (kcal/kg/d)	28 ± 8	23 ± 6	<0.0001
Protein (g/kg/d)	1.9 ± 0.7	2.0 ± 0.6	0.0443
Carbohydrate (g/kg/d)	2.9 ± 1.0	2.0 ± 1.0	<0.0001
Fat (g/kg/d)	1.0 ± 0.3	0.7 ± 0.2	<0.0001

Data are expressed as the mean ± SD. *n* = 41. Legend: d—day, g—gram, kcal—kilocalorie, kg—kilogram.

**Table 3:** Body Composition.

	Pre	Post	*p*-Value Pre vs. Post	Change
DXA Body Weight (kg)	71.3 ± 13.3	70.2 ± 13.1	<0.0001	−1.3 ± 1.3
InBody Body Weight (kg)	71.6 ± 12.9	70.4 ± 12.8	<0.0001	−1.1 ± 1.3
*p*-value for DXA vs. InBody	0.1351	<0.0001		0.0543
DXA Fat-free mass (kg)	52.6 ±10.3	52.4 ± 10.1	0.5508	−0.1 ± 1.1
InBody Fat-free mass (kg)	55.5 ± 11.3	55.6 ± 11.0	0.5765	0.1 ± 1.3
*p*-value for DXA vs. InBody	<0.0001	<0.0001		0.2271
DXA Fat Mass (kg)	18.9 ± 6.3	17.8 ± 6.3	<0.0001	−1.1 ± 0.6
InBody Fat Mass (kg)	16.1 ± 6.3	14.8 ± 6.4	<0.0001	−1.2 ± 1.2
*p*-value for DXA vs. InBody	<0.0001	<0.0001		0.3622
DXA Body Fat Percent (%)	26.4 ± 6.4	25.2 ± 6.4	<0.0001	−1.1 ± 0.9
InBody Body Fat Percent (%)	22.5 ± 7.5	21.0 ± 7.5	0.0001	−1.5 ± 1.7
*p*-value for DXA vs. InBody	<0.0001	<0.0001		0.1783

Data are expressed as the mean ± SD. *n* = 41. Legend: kg—kilogram.
